# Application of Kolmogorov–Sinai’s Metric Entropy for the Analysis of Mechanical Properties in the Bending Test of Epoxy–Rubber–Glass Composites

**DOI:** 10.3390/ma17205079

**Published:** 2024-10-18

**Authors:** Norbert Abramczyk, Grzegorz Hajdukiewicz, Adam Charchalis, Daria Żuk

**Affiliations:** Faculty of Marine Engineering, Gdynia Maritime University, 81-225 Gdynia, Poland; n.abramczyk@wm.umg.edu.pl (N.A.); g.hajdukiewicz@wm.umg.edu.pl (G.H.); a.charchalis@wm.umg.edu.pl (A.C.)

**Keywords:** epoxy–rubber–glass composites, rubber recyclate, three-point bending test, Kolmogorov–Sinai metric entropy

## Abstract

The article presents an analysis of the results obtained during the three-point bending test for seven variants of epoxy rubber–glass composites manufactured according to innovative technology. Different contents of rubber recyclate (3, 5, and 7%) and different methods of distribution of the recyclate in the composite structure (1, 2, and 3 layers with a constant share of 5% of the recyclate) were used in the tested materials. To determine the stress values at which critical failures of the tested materials are initiated in the bending test, an analysis was carried out using the Kolmogorov–Sinai (*E_K-S_*) metric entropy calculations. The analysis results showed that for each of the above-mentioned variants of the tested epoxy–glass composites, the onset of critical changes occurring in the material structure occurs below the recorded values of the flexural strength *Rmg*. The decrease in the *Rmg_K-S_* value in relation to *Rmg* is different for different material variants and depends mainly on the % content of rubber recyclate and the amount and method of decomposition of rubber recyclate in the layers of the analyzed materials. The research showed that the introduction of rubber recyclate into the composition of composites has a positive effect on their strength properties. This process allows for the efficient use of hard to degrade waste and opens up the possibility of using the newly developed materials in many industrial sectors.

## 1. Introduction

Advances in the field of composite materials manufacturing make them play a very important role in the design and implementation of modern structures. Using a combination of different materials in a single structure can lead to properties that are not achievable with homogeneous materials. This makes composites attractive for many applications [1,2,3,4]. However, the creation of complex material structures in composites raises the bar for analyzing the behaviour of these materials under load.

The method of analyzing the results of destructive tests using metric entropy calculations has been used in various fields of materials science and mechanical engineering [5,6]. The application of this method is based on performing calculations on sets of experimental data obtained during strength tests, such as a static tensile test, with the simultaneous verification of the obtained results in comparison with another, independent test method, such as acoustic emission [7,8].

In 1959, Andrei Kolmogorov introduced the concept of metric entropy, which linked statistical mechanics with chaos theory. Kolmogorov–Sinaia metric entropy (*E_K-S_*) is designed to describe the dynamics of system change. Unlike classical entropy, *E_K-S_* is associated with energy movement toward its dispersion. *E_K-S_* stands for the amount of energy per unit of time and takes positive values that can rise and fall [9,10].

For example, *E_K-S_* entropy calculations for isotropic metal samples in a static tension test assume distinct, measurable changes in process dynamics at specific points during the tensile process. The first of such points occurring in the tensioned metal after exceeding the proportionality limit is a clearly perceptible and, at the same time, critical point separating the phase of elastic deformation from the phase of elastic–plastic deformation, i.e., *R_e_* or yield strength [11]. The results of metric entropy calculations made on data sets obtained during strength tests allow us to assess the degree of complexity of the structures of the tested materials. The high variability of the metric entropy value in the course of destructive testing of samples usually indicates a very high dynamics of the process, which can be interpreted as the existence of various processes that lead to the deterioration of material structures during loading. An analysis of the results of metric entropy calculations allows you to identify relevant patterns and relationships in the test data. This can include detecting cycles of load changes, periodic alterations in the material’s structure during fatigue tests, or identifying periodic strength fluctuations [12].

Kolmogorov–Sinai ’s metric entropy can be used to evaluate the stability and homogeneity of materials during endurance tests [13,14]. An analysis of entropy changes over time or depending on loading conditions can provide information about possible changes in the structure of the material that can affect its strength.

The use of metric entropy also makes it possible to compare the strength of different materials based on the analysis of their test data. Different metric entropy standards can be used to determine the similarities or differences in the behaviour of materials during endurance tests [15].

Metric entropy is an innovative analytical tool for interpreting strength test results. It allows one to understand the transformations taking place in the structure of materials under load and identify key features affecting their strength and resistance to damage [16].

This paper presents the application of Kolmogorov–Sinai’s metric entropy (*E_K-S_*) to analyze the results obtained in the bending test of epoxy–rubber–glass composites. The study aimed to determine how the *E_K-S_* method can be used to evaluate the mechanical properties of bent composites. Through the use of mathematical and statistical tools, it is possible to deepen the knowledge about the behaviour of complex structures of composite materials under load and to indicate potential areas for further research and development of this topic.

The flexural testing of composite materials is designed to evaluate their strength and mechanical properties, especially for future use [17,18]. It allows one to investigate how composites cope with bending loads, which is crucial to guaranteeing the safety and reliability of structures made of these materials. These tests make it possible to determine the flexural strength limits and the degree of deflection and provide valuable information on their fracture toughness.

A bending test performed on a material created using a new technology or innovative composition makes it possible to detect weak points in the composite, allowing engineers to make changes to the design or manufacturing process to increase its strength and durability.

Most studies on rubber-infused composites indicate that materials containing repurposed rubber could be appropriate for various application requiring higher strength parameters, i.e., tensile strength and impact strength [15,19,20]. However, there is a lack of detailed studies on epoxy–glass composites with an admixture of rubber from recycled car tires, which would examine the impact of such an additive on the bending strength of these composites. To verify the mechanical properties of the composite materials in question, it is essential to conduct full-strength tests along with the analysis and evaluation of the results obtained.

As part of the presented research, composites based on glass fibre and epoxy resin, enriched with the addition of rubber recyclate, were developed and manufactured using innovative technology. Rubber recyclates, thanks to their high elastic properties, can increase the elasticity of composites. Adding rubber products to the composition of a composite can improve its ability to absorb energy during bending loads, which in turn can increase fracture toughness [21,22,23]. However, the final effect of rubber recyclate on the flexural strength of epoxy composites depends on many factors, such as the type and amount of recyclate, the mixing method, the type of materials used, the parameters of the curing process, and the specific application requirements. Therefore, it is essential to conduct detailed research and testing to assess this impact.

## 2. Materials and Methods

### 2.1. Properties of the Rubber Recyclate Used

**Figure 1:**
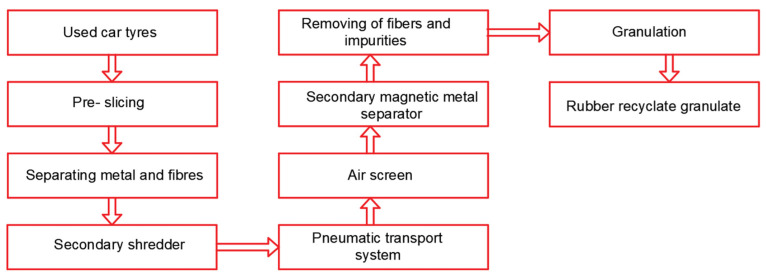
An example of a tire recycling process.

The rubber recyclate was produced by recycling used car tires. Tires are composed of various materials, including rubber, steel, and fabric. Figure 1 illustrates the flowchart of the rubber recycling process. The primary goal of rubber recycling is to reclaim and reuse these materials, minimizing waste and decreasing the demand for new raw materials.

To produce the research materials, Orzel-Base rubber recyclate (Orzel S.A., Poniatowa, Poland) from industrially processed car tires with a grain size of 0–3 mm was used as a modifier. The recyclate was screened using the laboratory sieve shaker LAB 11-200 (EKO-LAB, Jasien, Brzesko, Poland) in order to precisely separate its fraction. As an additive to the epoxy resin, a fraction of recyclate with a grain size of 0.5 mm to 1.5 mm was used in the composite. The percentage composition and physicochemical parameters of the rubber recyclate used in the tested materials are presented in Table 1 and Table 2.

### 2.2. Creation of Research Materials

**Figure 2:**
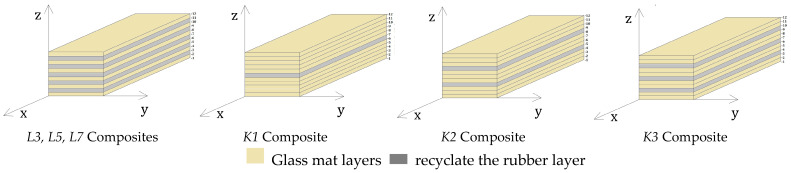
Diagram of the distribution of rubber recyclate layers in manufactured composites [25].

All designed material variants were created using EM 1002/300/125 construction glass mat (PHU Krisko, Lublin, Poland), which features a random fibre arrangement weighing 350 g/m^2^. The composite matrix utilized Epidian^®^6 epoxy resin with hardener Z-1 (SARZYNA CHEMICAL sp z o.o., Nowa Sarzyna, Poland). Technical parameters of the matrix are presented in Table 3. The research materials included *L3*, *L5*, and *L7* composites, which varied in the amount of recyclate mixed directly with the resin, and *K1*, *K2*, and *K3* composites that contained 5% recyclate, differing in their layering methods. The base material was a *K0* composite without any added recyclate. Figure 2 illustrates the application method of rubber recyclate within the composite structure. Detailed compositions of all composite variants are presented in Table 4.

The research materials were created using hand lamination, applying consistent double-sided pressure across all variants. The pressure value (675 N for one mould) was set to prevent excessive resin seepage and leakage. A rectangular steel mould measuring 300 mm × 900 mm and brushes and rollers were employed for the manual lamination process.

## 3. Results and Discussion

### 3.1. Bending Test

**Figure 3:**
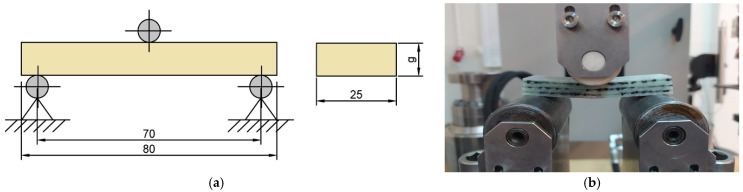
(**a**) Geometry and dimensions of specimens for three-point bending test (g—thickness of the sample); (**b**) a sample of *K3* material during a bending test.

The samples made of composite materials were prepared following the PN-EN ISO 14125:2001 standard [26]. They were cut from previously manufactured plates using waterjet cutting technology, which minimized the effect of temperature on the structure of the samples. The samples were then subjected to three-point bending tests using a Zwick Roell MPMD P10B testing machine with a hydraulic drive and TestXpert II 3.6.1 software (ZwickRoell Group, Ulm, Germany). The bending test was carried out by the pressure of the upper movable support with a force sensor; the deflection measurement was recorded automatically by analyzing the vertical displacement of the machine crosshead and processed and saved on the test bench with the TestXpert II 3.6.1 software. The bending test was carried out following the PN-EN ISO 14125:2001 standard. Figure 3 shows the geometry and dimensions of the samples.

The results of the basic analyses obtained as a result of the bending test are presented in the study [27]. The results of these measurements were used to carry out a further comparative analysis of the tested material variants in terms of strength parameters, which made it possible to assess the effect of rubber recyclate on these properties. They also allowed for a detailed analysis and the selection of material variants characterized by the best strength parameters.

### 3.2. Analysis of Metric Entropy of Mechanical Properties of Individual Material Samples

To assess the degree of homogeneity of the structure of the epoxy–rubber–glass composites studied, the Kolmogorov–Sinai metric entropy calculation method was used. This method made it possible to obtain model entropy value profiles for materials with different proportions of recyclate content (3, 5, and 7%) and different structures (1, 2, and 3 layers with 5% recyclate). The resulting entropy profiles were then compared with each other.

The method of calculating the K-S metric entropy is described by the authors in point 2.3 of Reference [14]. Although this study describes the study of a different type of composite using the results obtained in the static tensile test, the method of calculating the metric entropy *E_K-S_* is identical and based on the same programming tool “Entropy K-S”, program version 1.20 (Authors: Łukasz Mrugała—programmer, code author; Grzegorz Hajdukiewicz—functionality concept; Gdańsk, Poland 2021). In the publication mentioned above, the methodology of *E_K-S_* calculations is shown step by step on the example of one selected, 60-digit interval recorded during the tensile strain test ε, which was divided into six sub-intervals. The data and formulas used to calculate the *E_K-S_* are presented in Table 2 ([14]), while the result of the obtained entropy value is illustrated in Figure 7 ([14]) of the cited publication.

The comparison of waveform profile patterns of metric entropy calculated from experimental data sets allows for a deeper insight into the relationship between the structure and mechanical properties of the studied material variants. Thanks to this method, it is possible to understand the effect of rubber recyclate on the strength properties of epoxy–glass composites.

**Figure 4:**
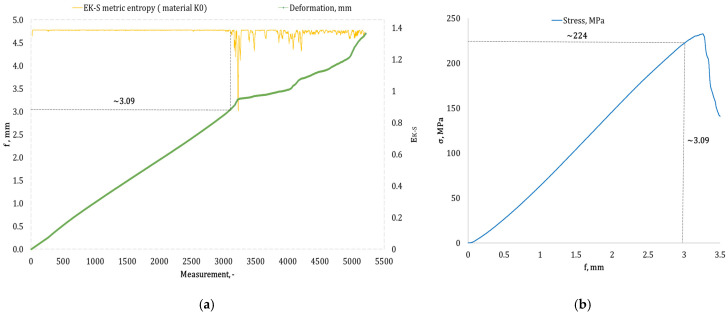
(**a**) Results of the metric entropy calculation *E_K-S_* as a function of test time for the *K0* composite sample; (**b**) bending curve for the *K0* composite sample, with the strain value obtained from the *E_K-S_* metric entropy calculations marked.

**Figure 5:**
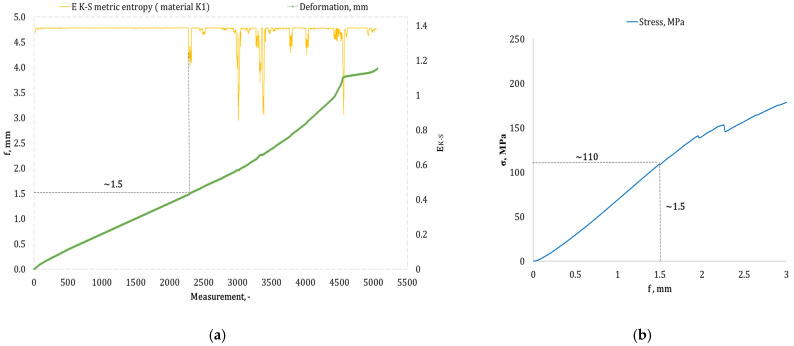
(**a**) Results of the metric entropy calculation *E_K-S_* as a function of test time for the *K1* composite sample; (**b**) bending curve for the *K1* composite sample, with the strain value obtained from the *E_K-S_* metric entropy calculations marked.

**Figure 6:**
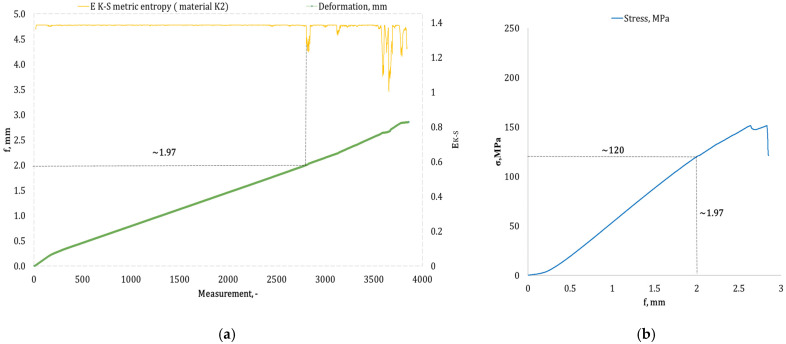
(**a**) Results of the metric entropy calculation *E_K-S_* as a function of test time for the *K2* composite sample; (**b**) bending curve for the *K2* composite sample, with the strain value obtained from the *E_K-S_* metric entropy calculations marked.

**Figure 7:**
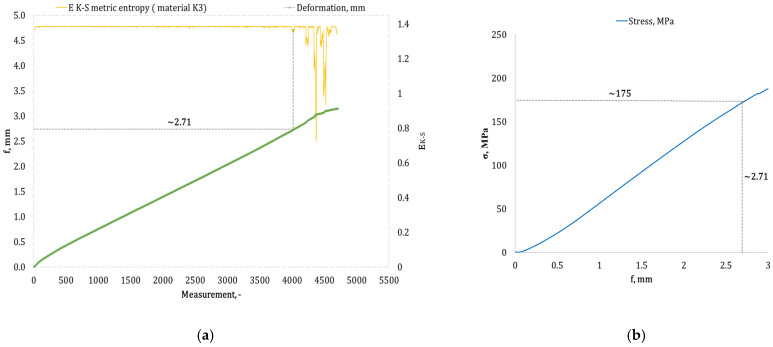
(**a**) Results of the metric entropy calculation *E_K-S_* as a function of test time for the *K3* composite sample; (**b**) bending curve for the *K3* composite sample, with the strain value obtained from the *E_K-S_* metric entropy calculations marked.

**Figure 8:**
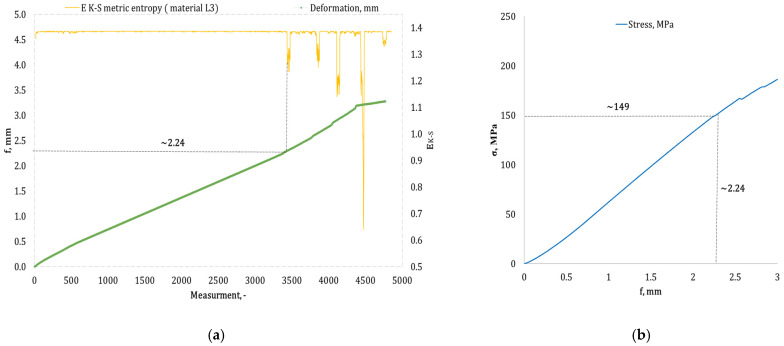
(**a**) Results of the metric entropy calculation *E_K-S_* as a function of test time for the *L3* composite sample; (**b**) bending curve for the *L3* composite sample, with the strain value obtained from the *E_K-S_* metric entropy calculations marked.

**Figure 9:**
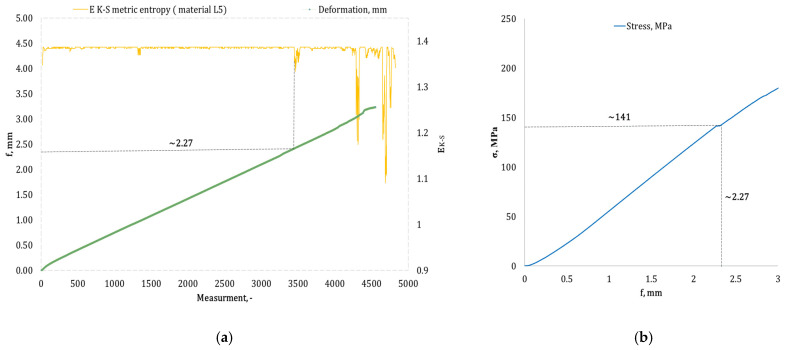
(**a**) Results of the metric entropy calculation *E_K-S_* as a function of test time for the *L5* composite sample; (**b**) bending curve for the *L5* composite sample, with the strain value obtained from the *E_K-S_* metric entropy calculations marked.

**Figure 10:**
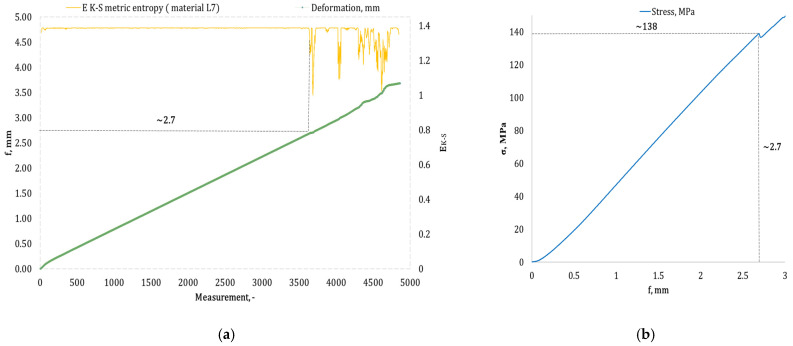
(**a**) Results of the metric entropy calculation *E_K-S_* as a function of the test time for the *L7* composite sample; (**b**) bending curve for the *L7* composite sample, with the strain value obtained from the *E_K-S_* metric entropy calculations marked.

Figure 4, Figure 5, Figure 6, Figure 7, Figure 8, Figure 9 and Figure 10 present selected bending curves for individual types of materials used in the research.

These drawings consist of two curves in the same variable (measurement point): the left vertical axis measures the course of the ε strain curve, while the right vertical axis measures the results of the calculation of the metric entropy *E_K-S_* as a function of time *ε = f*(*t*). The time on these graphs is represented by the number of sequential measurement points. All samples were tested using the same sampling method of the testing machine of 50 Hz, which means that one second of testing corresponded to 50 consecutive measurement points.

Calculations of metric entropy *E_K-S_* were carried out using sets of deflection values. *f* was recorded for each sample, maintaining the same length of the adopted intervals, i.e., 40 consecutive measurement points, and an equal number of sub-intervals, i.e., 4. For each variant of the composite material, the *E_K-S_* metric entropy change plot shows the characteristic point at which the first significant entropy decrease occurs. This local decrease in metric entropy indicates a significant change in the bending dynamics of a particular sample. The change in the dynamics of deformation (deflection) under the influence of a uniformly changing bending force (the test was carried out with a constant increase in force) should be understood as a violent response of the material. The occurrence of such a point indicates a critical quality change inside the composite, which may be related to the initiation of irreversible changes in the material, such as the onset of matrix cracks, delamination of composite fibres, and delamination of additives, including rubber recyclate.

After identifying the number of measuring points where there is a significant decrease in the metric entropy value *E_K-S_*, the deflection value *f_K-S_* for this point is read. Then, the *f_K-S_* value from Figure 4, Figure 5, Figure 6, Figure 7, Figure 8, Figure 9 and Figure 10 is transferred to the diagram *σ = f*(*t*) obtained from the testing machine, where the corresponding stress value *σ_K-S_* is read. Table 5 presents the mean values of Rmg and f obtained in the bending test and the mean values of deflections *f_K-S_* and the corresponding stress values *Rmg_K-S_* obtained using the Kolmogorov–Sinai metric entropy calculations *E_K-S_*.

By analyzing the data presented in Table 5, it is evident that for each type of epoxy–glass composite containing rubber recyclate, we are dealing with a decrease in the *f*-value of *K-S* in terms of the *f*-value. The same applies to the *Rmg_K-S_* value, which decreases compared to the values recorded in the *Rmg* test. It should, therefore, be acknowledged that incorporating rubber recyclate into the structure of epoxy–glass composites consistently reduces the material’s bending strength. However, the reduction in mechanical properties in the bending test is noticeably different depending on the percentage of recyclate and the way this recyclate is distributed inside the material structure. Thus, in the case of a 5% addition of recyclate placed in one layer in the composite structure, the highest value of reduction *f_K-S_* compared to *f* was recorded, amounting to 48.91%. This indicates the occurrence of critical structural damage to the sample material already at a deformation (deflection) of 1.4 mm. The deflection of 1.4 mm for this type of sample occurs with the average *Rmg_K-S_* of 108 MPa, which is a decrease of 31.64% compared to the *Rmg*. A selected sample from the group of single-layer ones marked as *K1* is shown in Figure 5a,b. The addition of 5% of rubber recyclate, distributed in the structure of the material in two layers (group designation *K2*), caused significant changes in the structure of the material at a deflection *f_K-S_* of 2.17 mm (*f* the average for this group of samples was 2.79 mm), which was a decrease of 22.22% and corresponded to the stress *Rmg_K-S_* of an average of 130 MPa (a decrease of 18.24% compared to the average *Rmg* 159 MPa). Figure 6a,b presents an example from this sample group. The distribution of 5% of rubber recyclate in the composite structure in three layers resulted in the results of average deflection *f_K-S_* of 2.70 mm (reduction relative to *f* by 10% from 3.16 mm). The average deformations *f_K-S_* corresponded to the bending stresses. *Rmg_K-S_* amounted to 171 MPa, i.e., 14.56% lower than *Rmg*, which amounted to 190 MPa. The *K3* sample group is shown in Figure 7a,b.

The addition of rubber recyclate in the amount of 3% placed in the structure of the material in a random form (a group of samples marked as *L3*) resulted in obtaining average results *f_K-S_* of 2.40 mm, which was a 21.05% decrease compared to the *f*-value of 3.04 mm. Average deformations *f_K-S_* corresponded to bending stresses *Rmg_K-S_* amounting to 152 MPa (lower by 16.48% than *Rmg* 182 MPa). An example sample from the *L3* group is shown in Figure 8a,b. Increasing the randomly distributed recyclate in the material structure to 5% resulted in the occurrence of critical changes in the structure of the obtained composite with a deflection *f_K-S_* of 2.58 mm (a decrease from 3.11 mm by 17.04%). The deflection *f_K-S_* occurred at *Rmg_K-S_*, amounting to 157 MPa (*Rmg* for this group of samples is 181 MPa). An example of a sample from the *L5* group is shown in Figure 9a,b. Another random increase in the share of recyclate in the composite structure to 7% (samples marked *L7*) caused the material to show significant qualitative changes from the deflection value *f_K-S_* of 2.62 mm (*f* the mean for this group was 3.20 mm). The mean values of *f_K-S_* corresponded to the mean values of *Rmg_K-S_*, amounting to 132 MPa, which was reduced by 12.58% compared to *Rmg*. This group of samples is represented by the example in Figure 10a,b.

Separate attention should be paid to the reference material, i.e., an epoxy–glass composite without the addition of rubber recyclate (sample group *K0*). This material showed a slight decrease in *f_K-S_* relative to *f*, i.e., a decrease from 3.29 mm to 3.16 mm, which was a decrease of 3.95%. The critical deflection values corresponded to the *Rmg_K-S_* stress of 234 MPa, reduced in relation to *Rmg* by only 0.43% from the value of 235 MPa.

In summary, the analysis of strength properties in the bending test, using metric entropy calculations for all seven composite types, revealed that the critical stress values (*Rmg_K-S_*), where structural changes in the composites occur, are lower than their bending strength (*Rmg*). This indicates that for every material tested, including the base material without rubber recyclate, the load limits in design and operation should be based on these lower critical stress values. Additionally, *E_K-S_* calculations showed that the incorporation of rubber recyclate significantly alters the elastic–plastic properties of the composite, with a decrease in *f_K-S_* observed in each case.

## 4. Conclusions

The data presented in Table 5 indicate that the strength properties of epoxy–rubber–glass and epoxy–glass sandwich composites, as measured in bending tests (such as *Rmg* and *f*), do not adequately characterize this type of material. This is especially true for composites modified with additives, as demonstrated by other research methods, including acoustic emission and optical techniques for measuring surface deformations. [7,8,27]. The calculations of the *E_K-S_* metric entropy performed on sets of deflection values obtained in the three-point bending test (the test takes place at a constant rate of force increase) allow us to better illustrate the dynamics of the material destruction process. We present the results of metric entropy calculations in a graphical form as *E_K-S_* = *f*(*t*), which allows for a broader assessment of the properties of this type of material. The use of *E_K-S_* calculations in the era of widespread use of powerful and fast computers allows us to capture the significant changes in the structure of the materials under study in a way that has not been available so far. Taking into account the percentages of the reduction in *Rmg_K-S_* in terms of *Rmg* and *f_K-S_* in terms of f, it should be recognized that in terms of bending strength in relation to the starting material, the three-layer composite, i.e., the K3 variant, deserves special attention. The *K3* variant of the composite also showed a small discrepancy in the strength parameters obtained in the bending test. Previous studies conducted by the authors showed that the variants of *K1*, *K2*, and *K3* composites have the most favourable properties compared to pure composite and with variants with a random distribution of recyclate between all composite layers (*L3*, *L5*, and *L7*). At the same time, the *K1*, *K2*, and *K3* composites showed favourable parameters in other types of strength tests, such as the static tensile test (K1 composite) and the impact test (*K2* composite). Due to the above, choosing a favourable variant depends on the potential application and determining which parameter should be crucial is possible.

The use of an innovative composite manufacturing technology allowed us to obtain materials with changed mechanical properties. The introduction of a modifier in the form of rubber recyclate as an additive to the polymer matrix not only contributes to the effective management of rubber waste but can also improve the vibration-damping properties and elasticity of the components due to the elastic properties of the modifier. In addition, further acoustic studies have shown a very beneficial effect of the addition of rubber recyclate on the sound insulation properties and the possibility of using the materials in question as partitions and sound insulation chambers [28].

Thanks to the combination of glass fibre, epoxy resin, and rubber recyclate, composites with unique properties have been obtained, which can contribute to the development of modern, ecological materials with a wide range of applications in various industrial sectors. Examples of potential applications may include the automotive, construction, marine, and aerospace industries. Epoxy–rubber–glass components can be used for the production of structural elements of vehicles, covers, wheel arches, and other components, where lightness, mechanical strength, and resistance to external factors, including vibrations, are required. Thanks to their high strength and corrosion resistance, these components can be used for the production of structural elements, façade panels, and composite reinforcement. Due to their resistance to water and corrosion, such composites can be used in the construction of boat hulls, yachts, or elements of marine installations. The lightness and strength of composites make them suitable for the production of structural elements in the aviation industry, which contributes to reducing the weight of aircraft and improving their fuel efficiency. Using composites in sports equipment, such as helmets, protectors, surfboards, or bicycles, can significantly increase the durability and comfort of these products.

## Figures and Tables

**Table 1:** Percentage composition of rubber recyclate [24].

Ingredient	Content, %
Styrene–butadiene rubber	20
Natural rubber	15
Silica	15
Soot	15
Butadiene rubber	10
Mineral and vegetable oils	10
Other (Zinc oxide, stearic acid)	6
Butyl and Halogenated butyl rubber	5
Sulphur	2
Resin	2

**Table 2:** Physical and chemical characteristics of recycled rubber utilized in materials [24].

Parameter	Value
Density	360–370 kg/m^3^
Flash point	>350 °C
Thermal decomposition	>180 °C

**Table 3:** Characteristics of epoxy resin Epidian^®^ 6.

Parameter	Unit	Value
Epoxy number	[Mole/100 g]	0.510–0.540
Density at 25 °C	[g/cm^3^]	1.17
Viscosity at 25 °C	[mPa·s]	1000–1500
Gel time 100 g at 20 °C	[min]	20
Curing time at 20 °C	[days]	7

**Table 4:** The mass percentage of the components in the tested composites.

Variant of Material	Arrangement Recyclate	Number of Layers of Glass Mat	Resin Content, %	Glass Mat Contents, %	Content Recyclate, %
*K0*	lack	12	60%	40%	0%
*K1*	1 layer	12	60%	35%	5%
*K2*	2 layers	12	60%	35%	5%
*K3*	3 layers	12	60%	35%	5%
*L3*	mixed with resin	12	60%	37%	3%
*L5*	mixed with resin	12	60%	35%	5%
*L7*	mixed with resin	12	60%	33%	7%

**Table 5:** Mean strength parameters are determined through bending tests, and average results are calculated using metric entropy.

Material	*f*, mm	*R_mg_*, MPa	*f_K-S_*_,_ mm	Error Bar *f_K-S_*_,_ mm	*Rmg_K-S_*, MPa	Error Bar *Rmg_K-S_*	Change in *f_K-S_* Value Relative to *f*, %	Change in *Rmg_K-S_*, Value Relative to *Rmg*, %
*K0*	3.29	235	3.16	2.98–3.30	234	218–249	−3.95	−0.43
*K1*	2.74	158	1.40	1.20–1.65	108	98–118	−48.91	−31.65
*K2*	2.79	159	2.17	1.97–2.55	130	107–145	−22.22	−18.24
*K3*	3.16	190	2.70	2.60–2.80	171	160–185	−14.56	−10.00
*L3*	3.04	182	2.40	1.93–2.56	152	131–180	−21.05	−16.48
*L5*	3.11	181	2.58	2.37–2.93	157	139–191	−17.04	−13.26
*L7*	3.20	151	2.62	2.45–2.70	132	119–145	−18.13	−12.58

## Data Availability

The data presented in this study are available on request from the corresponding author. The data are not publicly available due to its huge amount.
